# MonoPrior-Fusion: Monocular-Prior-Guided Multi-Frame Depth Estimation with Multi-Scale Geometric Fusion

**DOI:** 10.3390/s26020712

**Published:** 2026-01-21

**Authors:** Zhiwei Lin, Bohan Sun, Zhan Zhang, Linrui Qian, Nianyu Yi

**Affiliations:** 1 School of Mathematics and Computational Science, Xiangtan University, Xiangtan 411105, China; 202431510130@smail.xtu.edu.cn (Z.L.); yinianyu@xtu.edu.cn (N.Y.); 2 CogLeap.AI Space Intelligence (Wuxi) Technology Co., Ltd., Beijing 100080, China; sunbohan@cogleapai.com (B.S.); zhangzhan@cogleapai.com (Z.Z.)

**Keywords:** multi-frame depth estimation, monocular depth prior, geometric consistency, multi-scale fusion, multi-view stereo (MVS)

## Abstract

Precise 3D perception is critical for indoor robotics, augmented reality, and autonomous navigation. However, existing multi-frame depth estimation methods often suffer from significant performance degradation in challenging indoor scenarios characterized by weak textures, non-Lambertian surfaces, and complex layouts. To address these limitations, we propose MonoPrior-Fusion (MPF), a novel framework that integrates pixel-wise monocular priors directly into the multi-view matching process. Specifically, MPF modulates cost-volume hypotheses to disambiguate matches and employs a hierarchical fusion architecture across multiple scales to propagate global and local geometric information. Additionally, a geometric consistency loss based on virtual planes is introduced to enhance global 3D coherence. Extensive experiments on ScanNetV2, 7Scenes, TUM RGB-D, and GMU Kitchens demonstrate that MPF achieves significant improvements over state-of-the-art multi-frame baselines and generalizes well across unseen domains. Furthermore, MPF yields more accurate and complete 3D reconstructions when integrated into a volumetric fusion pipeline, proving its effectiveness for dense mapping tasks. The source code will be made publicly available to support reproducibility and future research.

## 1. Introduction

Depth estimation plays a central role in visual perception systems, enabling downstream tasks such as autonomous navigation, robotic interaction, and augmented reality. Recent advances in monocular depth estimation have achieved remarkable progress by leveraging large-scale datasets and powerful neural architectures [1,2,3,4,5]. However, monocular predictions inherently suffer from scale ambiguity and often become unreliable in low-texture or low-parallax regions where appearance cues provide insufficient geometric constraints [6].

Multi-view or multi-frame depth estimation, in contrast, grounds reconstruction on calibrated geometric relationships across views [7,8]. While this paradigm naturally alleviates scale ambiguity, its performance is strongly tied to photometric consistency. When the baseline is small, textures are weak, or repetitive patterns dominate the scene, feature correspondence becomes unstable, degrading the reliability of traditional cost-volume or plane-sweep matching. As a result, existing multi-frame pipelines struggle to maintain accurate and consistent depth in challenging real-world environments.

Despite recent efforts, three fundamental limitations persist in current multi-frame approaches. First, feature matching remains sensitive to the quality of photometric cues; small baselines or weak textures often cause ambiguous cost distributions even with multi-view aggregation [9,10]. Second, geometric consistency is typically enforced through local warping or single-plane evaluations, providing only limited global regularization and making predictions susceptible to noise. Third, although monocular depth priors contain rich structural information, prior works [11,12] generally incorporate them only as pseudo-labels or coarse regularizers, preventing these priors from influencing the core multi-view correspondence generation process.

To overcome these limitations, we propose MonoPrior-Fusion (MPF), a new multi-frame depth estimation framework that integrates pixel-wise monocular priors directly into multi-view matching. Unlike previous strategies that rely on monocular predictions only as auxiliary supervision or low-resolution initialization, MPF injects dense prior cues—depth, surface normals, and confidence—into the hypothesis space of the cost volume itself. This design enriches the matching evidence at a fine-grained level, enabling the network to disambiguate weak photometric cues and preserve structural details in low-texture or small-baseline scenarios.

Beyond prior-guided correspondence, MPF introduces a geometric consistency loss based on virtual planes that regularizes depth by enforcing agreement across a set of randomly sampled planes in 3D. This plane-level supervision complements local photometric and normal-based cues, providing stronger global geometric coherence than traditional warping-based constraints. To further enhance robustness across large depth ranges and complex indoor layouts, MPF employs a hierarchical fusion architecture across multiple scales that propagates geometric information from coarse global structure to fine spatial detail. Throughout the pipeline, MPF operates under known camera intrinsics and poses, following established practice in calibrated multi-view estimation.

**Figure 1:**
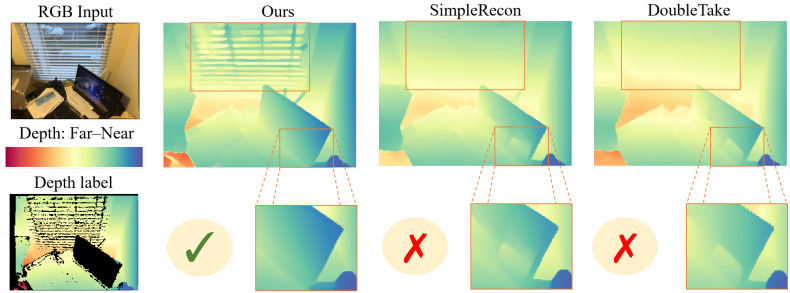
Qualitative comparison on ScanNetV2 [13]. Compared to SimpleRecon [8] and DoubleTake [14], MPF preserves sharper geometry in repetitive patterns (e.g., window blinds) and clearer depth boundaries at object edges (e.g., monitor). Note how baselines suffer from oversmoothing in these low-texture regions.

Figure 1 illustrates the advantage of our approach: MPF preserves sharper geometry and cleaner depth boundaries compared to purely photometric methods. To summarize, the main contributions of this work are as follows:

- We propose MonoPrior-Fusion (MPF), a novel framework that integrates pixel-wise monocular priors directly into the multi-view matching process to address ambiguities in low-texture scenarios.
- We design a hierarchical fusion architecture across multiple scales that propagates geometric cues from coarse to fine resolutions, improving structural coherence.
- We introduce a geometric consistency loss based on virtual planes that enforces plane-level alignment in 3D space, providing stronger global regularization.
- Extensive experiments on ScanNetV2, 7Scenes, TUM RGB-D, and GMU Kitchens demonstrate that MPF outperforms state-of-the-art methods and exhibits superior zero-shot generalization.

The remainder of this paper is organized as follows: Section 2 reviews related work. Section 3 details the MPF framework. Section 4 presents experimental results and ablation studies. Finally, Section 5 concludes this paper.

## 2. Related Work

### 2.1. Monocular Depth Estimation and Prior-Based Guidance

Monocular depth estimation has advanced significantly with the emergence of deep CNN- and transformer-based architectures. Early supervised methods [15,16] introduced multi-scale prediction pipelines that progressively refined feature representations, leading to consistent improvements in metric depth accuracy. Self-supervised approaches, such as Monodepth2 [17] and view-synthesis-based frameworks [18,19], further demonstrated that enforcing photometric consistency across adjacent frames provides a powerful supervisory signal, enabling depth learning without reliance on ground-truth depth annotations. More recently, large-scale pretrained models—including MiDaS [20], Depth Anything V2 [21], ZoeDepth [22], UniDepth [23], and Metric3Dv2 [24]—have shown strong generalization across diverse imaging conditions and camera settings, alongside architectural refinements that improve multi-scale feature utilization [25].

Monocular priors have also been incorporated into multi-view or multi-frame systems. Existing methods typically use monocular predictions to initialize cost volumes [11], refine sparse depth [26], or serve as auxiliary supervision [12,27]. However, these priors are usually injected only at late stages—as pseudo-labels, global regularizers, or coarse initialization—and therefore do not participate in early correspondence formation [28]. Consequently, the matching process remains largely photometric and often fails in textureless or low-parallax regions. To address this limitation, MPF integrates pixel-level monocular cues directly into the depth-hypothesis space, enabling finer structural discrimination during matching.

### 2.2. Learning-Based Multi-View Stereo and Geometric Consistency

Classical learning-based multi-view stereo (MVS) methods reconstruct metric depth by enforcing geometric consistency across calibrated views. MVSNet [7] introduced differentiable plane sweeping and learned cost-volume regularization, inspiring subsequent works such as CasMVSNet [10], DPSNet [29], and hybrid PatchMatch-based pipelines. Video-based extensions, including DeepVideoMVS [9] and SimpleRecon [8], adapt MVS principles to sequential frames, improving efficiency through temporally aligned features and lightweight correlation modules. More recently, MVSAnywhere [30] has demonstrated the potential of zero-shot MVS by leveraging large-scale pretraining, though often at a higher computational cost.

Despite these advances, cost-volume aggregation still relies heavily on local photometric consistency, making MVS vulnerable to small baselines, motion blur, low-texture regions, and repetitive patterns. Moreover, most methods enforce geometric consistency through local warping or single-plane evaluation, limiting their ability to incorporate global geometric constraints. In contrast, MPF stabilizes correspondence by augmenting the hypothesis space with monocular structural cues and introduces a geometric consistency loss based on virtual planes to enhance global regularization beyond local photometric signals.

### 2.3. Feed-Forward Multi-View Reconstruction

Recent feed-forward 3D reconstruction models aim to recover geometry without explicit triangulation or depth sweeping. DUSt3R [31] and MASt3R [32] estimate dense point maps or 3D correspondences directly from image pairs, while transformer-based architectures such as VGGT [33] jointly infer depth, camera parameters, and feature tracks through large-scale pretraining. These methods demonstrate impressive generalization and robustness to unconstrained viewpoints.

However, feed-forward models typically recover geometry only up to an unknown global scale, depend strongly on learned priors rather than explicit geometric reasoning, and often require joint processing of multiple images, which can be computationally demanding. Such properties limit their applicability in robotics or SLAM pipelines where metric, calibrated, and lightweight inference is required. MPF operates in a complementary setting: by leveraging calibrated camera geometry while injecting pixel-level monocular priors, it achieves metric depth estimation with improved robustness in challenging visual conditions.

Table 1 provides a comprehensive taxonomy of current paradigms. While pure monocular methods offer dense priors, they lack metric grounding, and feed-forward models often recover geometry only up to an unknown scale. Conversely, traditional MVS relies heavily on photometric consistency, making it vulnerable to textureless regions. Unlike existing prior-based MVS frameworks such as MVSAnywhere [30] that incorporate monocular cues as late-stage initialization or postprocessing, MPF implements a pixel-wise early fusion. By directly modulating the cost-volume hypothesis space with monocular priors (Equations (2)–(4)), MPF disambiguates matches at the source, effectively unifying the complementary strengths of both geometric and monocular paradigms.

## 3. Method

This section presents MPF, a framework for geometric fusion from multiple sources that integrates multi-frame matching, pixel-level monocular priors, multi-scale geometric reasoning, and consistency checks based on virtual planes. Traditional learning-based MVS systems rely heavily on photometric consistency and therefore degrade on weak textures and repetitive patterns or in small-baseline scenarios. Conversely, monocular depth models provide sharp structural priors but lack geometric grounding and metric scale.

As summarized in the taxonomy of Table 1, MPF unifies the complementary strengths of these two paradigms by implementing a pixel-wise early-fusion strategy. This is achieved through the following: (i) A multi-frame backbone that preserves camera geometry; (ii) A prior-guided modulation of depth hypotheses at the earliest stage of matching; (iii) Fusion volumes across multiple scales that propagate geometric cues across resolutions; (iv) Global 3D regularization via virtual planes.

**Figure 2:**
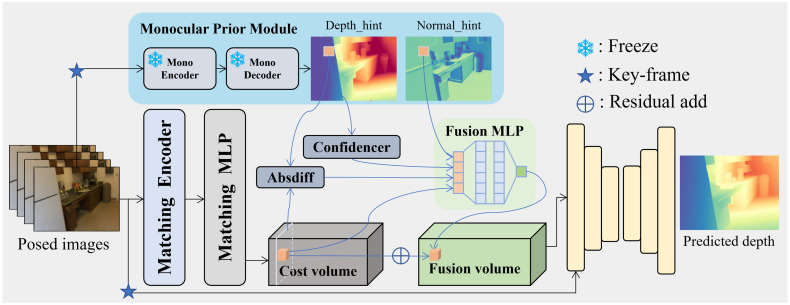
Overview of the proposed MPF framework. The Monocular Prior Module extracts pixel-wise cues—depth, confidence, and normal—from the input keyframe. These priors are fused with multi-frame matching features through a Fusion MLP, forming a hypothesis representation guided by priors that is further processed by the fusion volume and decoder to predict depth.

Together, these components enhance depth accuracy, robustness, and cross-domain generalization. Finally, the high-quality depth predictions produced by MPF serve as input to a volumetric fusion pipeline to enable dense 3D reconstruction. An overview of the entire MPF architecture is shown in Figure 2.

### 3.1. Multi-Frame Backbone

We adopt SimpleRecon (SR) [8] as the multi-frame matching backbone. SR aligns reference-frame features into the keyframe coordinate frame using known camera intrinsics and poses. For each depth hypothesis $d_{i}$, the warped reference features are correlated with keyframe features to form a 4D cost volume:

(1) $$C\in R^{C\times D\times H\times W},$$

where *C* denotes the feature channels, *D* the number of depth hypotheses, and (H,W) the spatial resolution.

A shared lightweight per-pixel MLP aggregates depth-dependent matching evidence for each pixel (x,y) and each hypothesis $d_{i}$, producing a scalar matching score $f_{d_{i}}\left(x,y\right)\in R$. MPF preserves the cost-volume construction of SR, including the geometric warping and the *D* discretized depth planes, while following a causal-view protocol to maintain geometric consistency.

### 3.2. Pixel-Wise Monocular Prior Fusion

Multi-view photometric matching becomes unreliable when texture or parallax is weak. To address this, MPF distinguishes itself from traditional late-fusion methods by incorporating pixel-wise monocular priors directly into the matching hypothesis space.

A pretrained Metric3Dv2 model [24] is applied to generate a monocular depth hint $\hat{d}$ and a surface-normal map $\hat{n}$. Additionally, a lightweight “Confidencer” module produces a reliability score $\hat{c}$ to downweight ambiguous monocular predictions. To achieve direct integration at the source of matching, we construct a 4-D hypothesis cue $h^{\left(0\right)}(i,x,y)$ for each depth hypothesis $d_{i}$ by concatenating the matching score with monocular geometric features:

(2) $$h^{\left(0\right)}\left(i,x,y\right)=\left[f_{d_{i}}\left(x,y\right),\;\left|\hat{d}\left(x,y\right)-d_{i}\right|,\;n\left(x,y\right),\;\hat{c}\left(x,y\right)\right],$$

where $|\hat{d}-d_{i}|$ measures the discrepancy between the prior and the hypothesis. A four-layer Fusion MLP M then maps this 4-D cue to a scalar:

(3) $${\tilde{f}}_{d_{i}}\left(x,y\right)=M\left(h^{\left(0\right)}\left(i,x,y\right)\right),$$

The final hypothesis response H(i,x,y) is obtained by a residual combination:

(4) $$H\left(i,x,y\right)={\tilde{f}}_{d_{i}}\left(x,y\right)+f_{d_{i}}\left(x,y\right).$$

Mechanistically, this formulation enables the network to leverage monocular structure to disambiguate matching. The prior discrepancy term acts as a soft geometric gate, suppressing hypothesis peaks that contradict the monocular hint. By modulating the hypothesis space directly, MPF ensures a geometry-aware representation that filters out photometric noise in challenging environments.

### 3.3. Multi-Scale Fusion Volume

**Figure 3:**
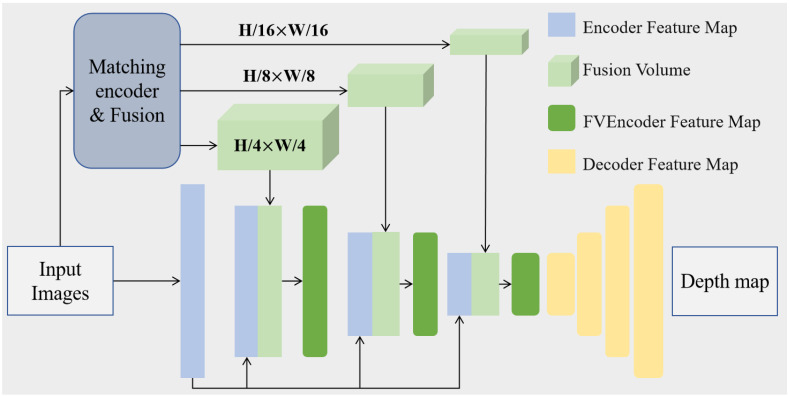
Architecture of the multi-scale fusion volume used in MPF. Fusion volumes at three spatial resolutions propagate geometric cues from coarse to fine scales, combining them with encoder features to produce a depth representation with improved structural consistency.

Indoor scenes often exhibit large depth variations, yet SR constructs its cost volume only at $\frac{H}{4}\times \frac{W}{4}$ spatial resolution, limiting the available global context. MPF introduces a hierarchical architecture for fusion volumes (FVs) that aggregates geometric cues across three resolutions, as illustrated in Figure 3.

The high-resolution FV is initialized by combining the backbone matching features (Section 3.1) with the prior-fused hypothesis responses from Section 3.2. This FV is then downsampled to generate mid- and low-resolution FVs, while maintaining a consistent depth discretization (D=64 hypotheses) across all scales. The three spatial resolutions,

$$\frac{H}{4}\times \frac{W}{4},\;\frac{H}{8}\times \frac{W}{8},\;\frac{H}{16}\times \frac{W}{16},$$

capture fine-grained details, mid-level structural layout, and coarse global geometry.

At each scale, the FV is refined by a residual BasicBlock [34]. Let $X_{\mathrm{fv}}^{\left(i\right)}$ denote the FV at scale *i* and let $F^{\left(i\right)}$ be the corresponding encoder feature map at the same spatial resolution. Following the coarse-to-fine structure shown in Figure 3, the FV from the previous coarser scale is first upsampled and fused with the current FV and its encoder features:

(5) $$S^{\left(i\right)}=B^{\times 2}\;\left(B\;\left(X_{\mathrm{fv}}^{\left(i\right)}⊕\mathrm{US}\left(S^{\left(i-1\right)}\right)\right)⊕F^{\left(i\right)}\right),\;i>1,$$

where B denotes a single BasicBlock, $B^{\times 2}$ applies two consecutive BasicBlocks, ⊕ denotes channel-wise concatenation, and error denotes bilinear upsampling. At the finest scale, the coarse feature does not exist and the refinement reduces to

(6) $$S^{\left(1\right)}=B^{\times 2}\;\left(B\left(X_{\mathrm{fv}}^{\left(1\right)}\right)⊕F^{\left(1\right)}\right).$$

This hierarchical fusion allows coarse-scale structure to guide finer refinements while preserving sharp depth boundaries. By propagating geometric cues across resolutions, MPF improves both global consistency and fine-grained structural accuracy.

### 3.4. Geometric Regularization

MPF enhances geometric fidelity through two complementary constraints, which operate at different spatial scales to improve depth consistency.

#### 3.4.1. Regularization Based on Surface Normals

Given a predicted depth map z(u,v), we estimate local geometric slopes using Sobel filters (3×3). The resulting depth gradients $\left(d_{x},d_{y}\right)$ are mapped to a 3D vector under the pinhole model:

(7) $$\begin{array}{c} n_{x}\left(u,v\right)=\frac{d_{x}f_{x}}{z(u,v)},\;n_{y}\left(u,v\right)=\frac{d_{y}f_{y}}{z(u,v)},\;n_{z}\left(u,v\right)=1, \end{array}$$

where setting $n_{z}=1$ follows the small-angle approximation [35]. This vector is then normalized to obtain the predicted unit surface normal:

(8) $$\hat{n}\left(u,v\right)=\frac{\left(n_{x}(u,v),n_{y}(u,v),n_{z}\left(u,v\right)\right)}{∥\left(n_{x}(u,v),n_{y}(u,v),n_{z}\left(u,v\right)\right){∥}_{2}}.$$

Ground-truth normals n(u,v) are either provided by the dataset or computed from ground-truth depth using the same procedure. The surface-normal loss penalizes angular deviation via

(9) $$L_{\mathrm{sn}}=\frac{1}{\left|V\right|}\sum_{(u,v)\in V}\left(1-|\hat{n}\left(u,v\right)\cdot n\left(u,v\right)|\right),$$

where V denotes the set of valid pixels.

#### 3.4.2. Consistency Based on Virtual Planes

While $L_{\mathrm{sn}}$ enforces local smoothness, it remains sensitive to noise. To introduce a more global geometric constraint, we adapt virtual-normal supervision [35] to the multi-view depth setting by enforcing consistency over randomly sampled 3D planes.

For a pixel $\left(u_{k},v_{k}\right)$ with depth $d_{k}$, back-projection into 3D yields

(10) $$a_{k}=\frac{d_{k}\left(u_{k}-u_{c}\right)}{f_{x}},\;b_{k}=\frac{d_{k}\left(v_{k}-v_{c}\right)}{f_{y}},\;c_{k}=d_{k},$$

where $\left(u_{c},v_{c}\right)$ denotes the principal point. We sample non-degenerate triplets $T={\left\{{\left(Q_{A},Q_{B},Q_{C}\right)}_{k}\right\}}_{k=1}^{M}$ within the keyframe only to avoid pose-induced noise. Each triplet satisfies

(11) $$\delta \leq ∠\left(\vec{Q_{A}Q_{B}},\vec{Q_{A}Q_{C}}\right)\leq \gamma ,\;|\vec{Q_{A}Q_{B}}\left|,\right|\vec{Q_{A}Q_{C}}|>\epsilon .$$

Using ground-truth depth, each triplet defines a plane normal:

(12) $$N_{k}=\frac{\left(\vec{Q_{B}Q_{A}}\right)\times \left(\vec{Q_{B}Q_{C}}\right)}{∥\left(\vec{Q_{B}Q_{A}}\right)\times \left(\vec{Q_{B}Q_{C}}\right){∥}_{2}}.$$

Applying the same construction to the predicted depth yields the estimated normal ${\hat{N}}_{k}$. The geometric consistency loss based on virtual planes is defined as follows:

(13) $$L_{\mathrm{vn}}=\frac{1}{\left|T\right|}\sum_{k\in T}\left(1-|{\hat{N}}_{k}\cdot N_{k}|\right).$$

Compared with purely local normal supervision, $L_{\mathrm{vn}}$ enforces geometric alignment over larger spatial regions by constraining entire planar surfaces in 3D. Crucially, this global constraint helps rectify scale ambiguities often present in monocular priors, ensuring that the fused depth map respects the structural layout of the scene while preserving sharp discontinuities.

### 3.5. Training Objective

MPF is trained using a combination of depth, gradient, multi-frame consistency, and geometric regularization losses. The overall objective follows the training protocol of SimpleRecon (SR) [8], while incorporating our proposed constraints derived from surface normals and virtual planes.

#### 3.5.1. Baseline Losses

Following SR, we supervise the predicted depth map using three standard losses:

- Depth regression loss $L_{\mathrm{depth}}$, implemented as an $\ell_{1}$ loss on inverse depth, identical to SR.
- Gradient loss $L_{\mathrm{grad}}$, computed using first-order finite differences on depth to encourage edge alignment.
- Multi-view consistency loss $L_{\mathrm{mv}}$, following SR’s formulation based on reprojection depth consistency between the reference views and the predicted keyframe depth.

These baseline terms encourage photometric and geometric agreement across the multi-frame backbone.

#### 3.5.2. Proposed Geometric Losses

The surface-normal loss $L_{\mathrm{sn}}$ (Section 3.4) provides local geometric refinement and improves high-frequency details. The consistency loss based on virtual planes $L_{\mathrm{vn}}$ enforces global geometric alignment by encouraging predicted 3D points to agree with ground-truth planar structures.

Together, $L_{\mathrm{sn}}$ and $L_{\mathrm{vn}}$ provide complementary local and global geometric constraints.

#### 3.5.3. Total Loss

The full training objective is

(14) $$L=L_{\mathrm{depth}}+L_{\mathrm{sn}}+\alpha_{\mathrm{grad}}L_{\mathrm{grad}}+\alpha_{\mathrm{mv}}L_{\mathrm{mv}}+\alpha_{\mathrm{vn}}L_{\mathrm{vn}},$$

where we follow SR and set $\alpha_{\mathrm{grad}}=1.0$ and $\alpha_{\mathrm{mv}}=0.2$. The weight for the loss based on virtual planes is $\alpha_{\mathrm{vn}}=0.2$. All losses are computed only at the final output resolution and normalized by the number of valid pixels.

This combination jointly supervises fine-scale local geometry, cross-view photometric reasoning, and global structural consistency, consistent with the design for geometric fusion from multiple sources of MPF.

### 3.6. Implementation Details

We follow the causal-view training protocol of SR [8], where each keyframe aggregates seven preceding reference frames sampled with stride 1. MPF is trained for 20 epochs using AdamW with an initial learning rate of $1\times 10^{-4}$. All remaining optimization and hardware configurations—including batch sizes, learning rate scheduling, and memory management strategies—are provided in Appendix A. A detailed analysis of computational efficiency (runtime and memory) is presented in Section 4.5.

## 4. Experiments

This section evaluates MPF on large-scale indoor RGB-D datasets, examining depth accuracy, cross-domain generalization, ablation behaviors, 3D reconstruction quality, and computational efficiency. To maintain clarity, complete metric definitions, extended qualitative comparisons, and additional tables are provided in the Appendix B.

### 4.1. Datasets and Evaluation Protocol

MPF is trained exclusively on ScanNetV2 [13] and evaluated in a strictly *zero-shot* manner on 7Scenes [36], TUM RGB-D [37], and GMU Kitchens [38]. These datasets exhibit diverse challenges, including low texture, repetitive indoor patterns, motion blur, strong lighting variation, and high-resolution imagery. Dataset statistics are summarized in Table 2.

We report the standard depth metrics AbsRel, AbsDiff, SqRel, and δ-accuracy thresholds. Full definitions are available in Appendix B. Invalid or missing ground-truth depth pixels are masked out following common RGB-D evaluation protocols.

We compare MPF against representative multi-view and multi-frame methods: DPN [29], DELTAS [39], GPMVS [40], DVMS [9], SimpleRecon (SR) [8], DoubleTake (DT) [14], and MVSA [30]. This selection covers both established baselines and the most recent state-of-the-art approaches.

### 4.2. Results on ScanNetV2 (Seen Domain)

**Figure 4:**
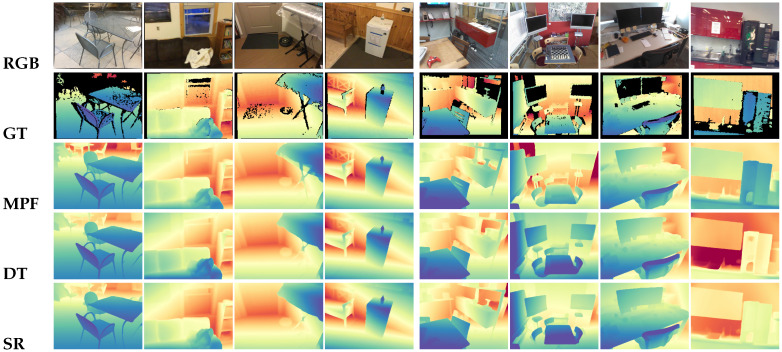
Qualitative depth comparisons on samples from ScanNetV2 and 7Scenes [13,36]. Cool colors (e.g., blue) indicate closer distances, while Warm colors (e.g., red) indicate further distances. MPF produces clearer geometric boundaries and more consistent depth than SR and DT, especially in low-texture regions.

Table 3 summarizes the quantitative evaluation on the ScanNetV2 test set. MPF achieves state-of-the-art performance, outperforming both the strong baseline SimpleRecon (ECCV 2022) and the recent DoubleTake (ECCV 2024). Specifically, compared to SimpleRecon, MPF reduces AbsRel by 11% and SqRel by approximately 35%. This significant improvement validates that our proposed prior-guided modulation effectively disambiguates cost volumes in regions where photometric consistency alone fails (e.g., white walls and reflective floors), preventing the “oversmoothing” artifacts common in traditional multi-frame methods. Qualitative comparisons in Figure 4 further confirm that MPF recovers sharper object contours and thinner structures.

### 4.3. Zero-Shot Generalization to 7Scenes, TUM, and GMU

To evaluate robustness, we test models trained on ScanNetV2 directly on unseen datasets without fine-tuning.

7Scenes. As shown in Table 4 (left), MPF achieves the best performance across all metrics on 7Scenes. Since 7Scenes contains extremely low texture and repetitive patterns (e.g., stairs and cabinets), purely photometric methods like SR and DT struggle to find reliable correspondence. By leveraging monocular structural priors, MPF effectively rectifies these ambiguities, preserving planar structures and depth discontinuities even in textureless regions.

TUM RGB-D. Results on the large-scale TUM dataset are shown in Table 4 (right). It is worth noting that MVSA [30] achieves high δ-accuracy ($a_{10}$) due to its large-scale pretraining, which learns robust relative depth. However, MVSA suffers from scale ambiguity in zero-shot transfer, resulting in higher absolute errors (AbsDiff). In contrast, MPF delivers superior metric accuracy (lowest AbsDiff and AbsRel), demonstrating that our framework successfully grounds monocular cues with multi-view geometry to maintain precise metric scale.

**Figure 5:**
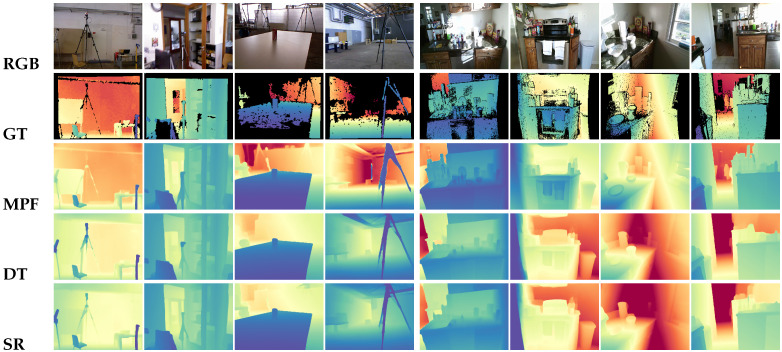
Qualitative depth comparisons on TUM and GMU datasets [37,38]. Cool colors (e.g., blue) indicate closer distances, while Warm colors (e.g., red) indicate further distances. MPF produces more stable and complete depth maps than SR and DT, recovering clearer scene geometry under challenging conditions such as motion blur (TUM) and strong lighting variation (GMU).

GMU Kitchens. GMU presents extreme challenges with dramatic lighting variation and reflective surfaces. As shown in Table 5, classical baselines exhibit catastrophic failure modes (high AbsDiff > 0.27) due to unreliable photometric cues. In contrast, MPF maintains stable performance (AbsDiff ≈ 0.09), substantially outperforming all baselines. Note that we report only stable multi-frame results; MVSA produced unstable predictions on this dataset due to the severe domain shift and sensor noise and is thus excluded from this specific comparison (raw outputs provided in Appendix B). Qualitative visualizations in Figure 5 show that MPF produces clean depth on strongly lit surfaces where other methods exhibit noisy or fragmented geometry.

### 4.4. Ablation and Component Analysis

Ablation experiments are performed on the 7Scenes dataset using a 40% randomly sampled subset of the ScanNetV2 training data. This reduced subset accelerates experimentation while maintaining a representative distribution of indoor scenes.

#### 4.4.1. Component Effectiveness

Table 6 summarizes the effect of progressively adding each MPF component. The baseline configuration (MPF-base w/o F,M,V) preserves only the multi-frame matching backbone. Given that the baseline is architecturally equivalent to SR, the qualitative comparisons in Figure 4 and Figure 5 serve as a direct visual ablation, demonstrating the cumulative geometric gains achieved by our proposed prior-guided fusion and consistency constraints over the pure photometric baseline.

As shown in Table 6, the baseline exhibits the lowest accuracy (AbsRel 0.0630), reflecting the inherent limitations of purely photometric correspondence. Incorporating the fusion module (w/F) integrates monocular priors directly into the cost volume, which noticeably improves depth hypothesis discrimination. Adding the hierarchical fusion volume (w/F,M) further strengthens geometric reasoning by enabling coarse-to-fine feature aggregation. Finally, the full model (w/F,M,V) achieves the highest accuracy, demonstrating that the geometric consistency loss based on virtual planes provides complementary global geometric constraints. Visually, this corresponds to the recovery of sharp planar structures and fine edges in MPF compared to the oversmoothed baseline results (SR) in Figure 4.

#### 4.4.2. Cost-Volume Discriminability

To verify that our method improves the quality of the matching distribution itself (and not just the final regression), Table 7 evaluates Rank Percentile (RP), Depth Margin (DM), and Quadfit MAE (Q-MAE) before and after applying the Fusion Network. Remarkably, the fusion module reduces the Rank Percentile error by over 5× at the 1/4 resolution (0.354 → 0.051). This provides direct quantitative evidence of how our pixel-wise early fusion manifests in practice: by modulating the hypothesis space at the source (as described in Section 3.2), MPF effectively “sharpens” the probability distribution around the true depth. This structural improvement enables the network to recover correct geometry even when photometric signals are ambiguous, which is a direct consequence of our proposed direct integration strategy.

### 4.5. Depth Reconstruction and Runtime Analysis

**Figure 6:**
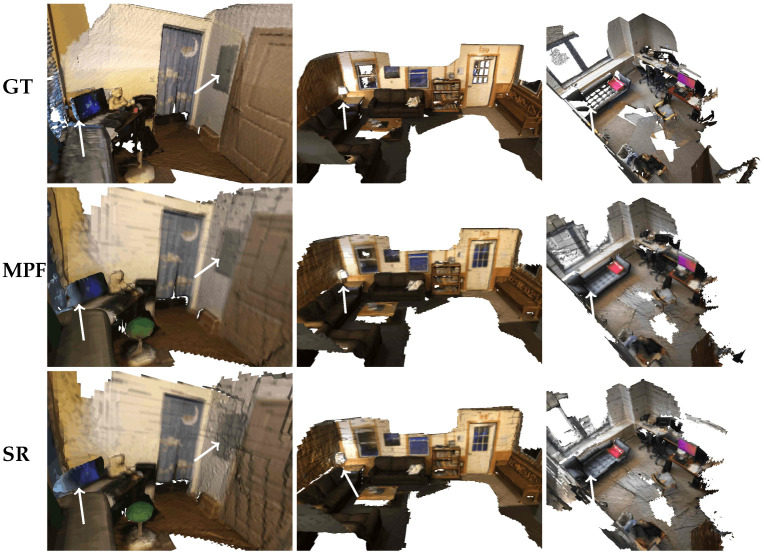
Qualitative 3D reconstruction results on ScanNetV2 [13]. The highlighted regions (white arrows) show that MPF reconstructs smoother wall surfaces, preserves fine geometric details, and recovers large indoor structures more completely than SR.

We integrate predicted depths into a volumetric fusion pipeline following TransformerFusion [41]. As shown in Table 8, MPF achieves the lowest geometric error and highest precision. Figure 6 illustrates that MPF reconstructs smoother planar surfaces, cleaner object boundaries, and finer geometric structures than SR, confirming the benefit of more accurate depth inputs for downstream 3D reconstruction.

#### Computational Efficiency

MPF processes each keyframe in 0.172 s on NVIDIA GeForce RTX 4090 GPU (NVIDIA Corporation, Santa Clara, CA, USA), of which 0.047 s is spent on the frozen monocular prior and 0.114 s on cost-volume construction and fusion. Peak memory consumption is 6.4 GB. With a 10:1 keyframe compression ratio, MPF achieves throughput compatible with real-time multi-frame pipelines.

### 4.6. Limitations and Discussion

While monocular priors significantly improve geometric detail, they introduce additional inference cost (approx. 47 ms) and may be sensitive to strong domain shifts where the pretrained prior fails. MPF also assumes known intrinsics and poses. Recent pose-free frameworks [32,33] suggest promising directions for removing this requirement. Future work will explore more efficient prior integration and pose-free multi-view depth estimation.

## 5. Conclusions

We presented MonoPrior-Fusion (MPF), a multi-frame depth estimation framework that combines monocular priors with multi-view geometric reasoning. A fusion module across multiple scales and a geometric consistency loss based on virtual planes were introduced to address textureless regions, small baselines, and challenging indoor conditions. Extensive experiments demonstrate that MPF achieves strong accuracy, robustness, and practical runtime efficiency across multiple benchmarks. These results highlight MPF’s potential for deployment in real-world 3D perception, reconstruction, and embodied AI systems. Future work will extend MPF to outdoor and dynamic environments and investigate integration with large-scale pretrained vision models.

## Figures and Tables

**Table 1:** Taxonomy and comparison of MPF with representative depth estimation paradigms. We explicitly distinguish our early-fusion mechanism, which modulates the hypothesis space at a pixel-wise level, from traditional late-fusion or pure photometric MVS methods.

Method Category	Representative Methods	Mechanism (Implementation Details)			Key Properties	
		Integration Stage	Fusion Granularity	Matching Guidance	Metric Scale	Texture Sensitivity
Pure Monocular	Metric3Dv2 [24], DepthAnything [21]	Direct Prediction	Pixel-wise	Learned Prior	Relative	Low
Feed-forward Recon.	DUSt3R [31], VGGT [33]	Implicit/Learned	Feature-level	Global Context	Unknown/Rel.	Low
Traditional MVS	SimpleRecon [8]	None	N/A	Photometric Only	Metric	High
**Prior-based MVS**	MVSAnywhere [30]	Late/Auxiliary	Image-level	Feature Refinement	Metric	Medium
**Ours (MPF)**	**Proposed Framework**	**Early (Cost Vol.)**	**Pixel-wise**	**Prior-modulated**	**Metric**	**Low**

**Table 2:** Overview of the datasets used in our experiments. Each dataset exhibits distinct challenges that influence multi-frame depth estimation performance.

Dataset	Scenes	Characteristics
ScanNetV2	1513	General indoor environments
7Scenes	13	Low texture, Repetitive patterns
TUM	13	Large-scale areas, Motion blur
GMU	9	Lighting variation, High resolution

**Table 3:** Depth evaluation on ScanNetV2 [13]. We compare against representative multi-frame MVS models and recent SOTA methods. Best and second-best results are shown in **bold** and underline. Arrows (↑/↓) indicate that higher/lower values are better.

Method	AbsDiff ↓	AbsRel ↓	SqRel ↓	$a_{10}\;↑$	$a_{25}\;↑$
DPN	0.1550	0.0794	0.0299	73.55	93.27
DELTAS	0.1498	0.0787	0.0276	73.65	93.77
GPMVS	0.1619	0.0823	0.0346	73.04	92.62
DVMS	0.1181	0.0579	0.0189	83.82	96.78
SR	0.0869	0.0429	0.0127	90.85	98.06
DT	0.0766	**0.0372**	0.0115	93.16	98.31
**MPF**	**0.0721**	0.0380	**0.0082**	**93.17**	**98.89**

**Table 4:** Zero-shot depth evaluation on 7Scenes [36] and TUM [37] using models trained only on ScanNetV2. Best and second-best results are shown in **bold** and underline. Arrows (↑/↓) indicate that higher/lower values are better.

	7Scenes (Zero-Shot)					TUM (Zero-Shot)				
Method	AbsDiff ↓	AbsRel ↓	SqRel ↓	$a_{10}$↑	$a_{25}$↑	AbsDiff ↓	AbsRel ↓	SqRel ↓	$a_{10}$↑	$a_{25}$↑
DPN	0.1793	0.0985	0.0406	65.51	89.95	0.7950	0.1832	0.3450	46.32	67.71
DELTAS	0.1847	0.1037	0.0406	62.19	89.94	0.9964	0.2292	0.4778	36.84	57.48
GPMVS	0.1749	0.0942	0.0428	68.19	91.00	0.7869	0.1866	0.3328	43.32	66.96
DVMS	0.1280	0.0684	0.0211	78.73	95.79	0.8689	0.1882	0.4018	47.46	66.66
SR	0.1048	0.0575	0.0157	84.60	97.30	0.9301	0.1981	0.4350	46.24	63.41
DT	0.0993	0.0536	0.0161	87.38	96.77	0.9672	0.2037	0.4890	48.36	63.34
MVSA	0.1214	0.0661	0.0274	81.85	95.06	0.5673	0.1461	0.3649	**63.97**	**83.14**
**MPF**	**0.0915**	**0.0511**	**0.0108**	**87.94**	**98.37**	**0.5376**	**0.1411**	**0.1567**	46.65	77.37

**Table 5:** Depth evaluation on GMU Kitchens [38]. Baseline methods (SR and DT) degrade significantly under strong lighting variations, whereas MPF maintains robustness. Unstable predictions from MVSA are excluded. Best and second-best results are shown in **bold** and underline. Arrows (↑/↓) indicate that higher/lower values are better.

Method	AbsDiff ↓	AbsRel ↓	SqRel ↓	$a_{10}$↑	$a_{25}$↑
DELTAS	0.5585	0.5853	0.5713	14.44	33.43
DVMS	0.5966	0.5961	0.6262	13.71	31.77
SR	0.3261	0.3332	0.1782	21.43	49.81
DT	0.2735	0.2549	0.1195	27.97	59.48
**MPF**	**0.0905**	**0.0711**	**0.0156**	**75.73**	**95.47**

**Table 6:** Ablation study on 7Scenes [36]. “F”, “M”, and “V” correspond to the fusion module, multi-scale fusion, and virtual-plane loss. Each component contributes to the progressive improvement in depth accuracy. Best results are shown in **bold**. Arrows (↑/↓) indicate that higher/lower values are better.

Method	AbsRel ↓	SqRel ↓	RMSE ↓	$a_{10}$↑	$a_{25}$↑
MPF-base (w/o F,M,V)	0.0630	0.0181	0.1737	82.15	96.69
MPF-base (w/F)	0.0615	0.0144	0.1563	82.23	97.86
MPF-base (w/F,M)	0.0583	0.0131	0.1509	83.54	98.08
**MPF (w/F,M,V)**	**0.0573**	**0.0129**	**0.1498**	**84.17**	**98.20**

**Table 7:** Cost-volume quality on 7Scenes before and after applying the Fusion Network. Lower values indicate sharper and more accurate matching distributions.

Resolution	Status	RP ↓	DM ↓	Q-MAE ↓
1/4	Before	0.354	2.91	2.72
	After	0.051	0.14	0.14
1/8	Before	0.139	0.40	0.39
	After	0.114	0.33	0.32
1/16	Before	0.381	2.66	2.46
	After	0.067	0.19	0.19
Overall	Before	0.313	2.41	2.25
	After	0.064	0.18	0.18

**Table 8:** Mesh reconstruction evaluation on ScanNetV2 [13]. Best and second-best results are shown in **bold** and underline. Arrows (↑/↓) indicate that higher/lower values are better.

Method	Acc ↓	Chamfer ↓	Prec ↑	Recall ↑
DELTAS	11.95	9.71	0.478	0.533
ESTDepth	12.71	10.12	0.456	0.542
DVMS	10.68	8.79	0.541	0.592
NeuralRecon	5.09	7.11	0.630	0.612
SR	5.53	5.81	0.686	0.658
DT	4.70	5.09	0.730	**0.701**
MVSA	4.93	5.66	0.616	0.696
**MPF**	**4.23**	**4.90**	**0.752**	0.650

**Figure A1:**
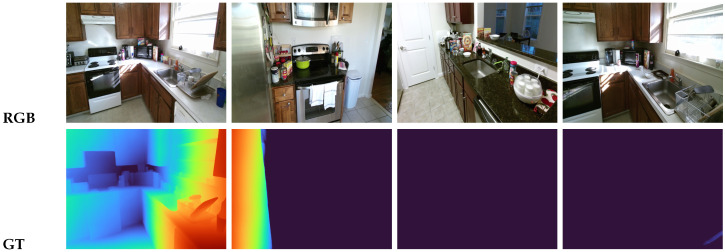
Qualitative MVSA results on GMU. Warm colors (e.g., red) indicate closer distances, while cool colors (e.g., blue) indicate further distances. In a few cases, MVSA predicts reasonable depth, but in most scenes the outputs collapse to uniform single-color maps, providing no useful geometric information. This confirms that MVSA is not suitable for GMU despite correct preprocessing.

## Data Availability

All data supporting the findings of this study are obtained from publicly available RGB-D datasets. The complete data preparation pipeline, experimental code, and evaluation scripts are publicly available at https://github.com/2k-lin/MPF-DepthEstimation (accessed on 18 January 2026), ensuring full reproducibility of the reported results.

## Abbreviations

The following abbreviations are used in this manuscript:

AbsDiff	Absolute Difference
AbsRel	Absolute Relative error
CNN	Convolutional Neural Network
DT	DoubleTake
FV	Fusion Volume
GMU	George Mason University (Dataset)
GT	Ground Truth
M3v	Metric3Dv2
MLP	Multi-Layer Perceptron
MPF	MonoPrior-Fusion
MVS	Multi-View Stereo
RGB-D	Red–Green–Blue and Depth
RMSE	Root Mean Square Error
SLAM	Simultaneous Localization and Mapping
SqRel	Squared Relative error
SR	SimpleRecon
TUM	Technical University of Munich (Dataset)

## Appendix A. Full Implementation Details

### Appendix A.1. Training Setup

All experiments follow the causal-view protocol of SimpleRecon (SR) [8], in which each keyframe is paired with seven temporally preceding reference frames. We train MPF for 20 epochs using AdamW with an initial learning rate of $1\times 10^{-4}$, decayed linearly. Two hardware configurations are used: (i) 40% of ScanNetV2 [13] with batch size 16 on two NVIDIA A100 GPUs (NVIDIA Corporation, Santa Clara, CA, USA) for ablation studies and (ii) full ScanNetV2 with batch size 8 on two NVIDIA GeForce RTX 4090 GPU (NVIDIA Corporation, Santa Clara, CA, USA) for final benchmarking.

### Appendix A.2. Depth Hypotheses and Multi-Scale Volumes

We adopt D=64 depth planes uniformly sampled in inverse depth over [0.001,10] meters. The same *D* hypotheses are used at all three spatial resolutions ($\frac{H}{4}$, $\frac{H}{8}$, and $\frac{H}{16}$), ensuring consistent geometric discretization. To control memory usage, feature channel dimensions decrease with spatial resolution, and coarse levels use proportionally fewer channels.

### Appendix A.3. Network Architecture

EfficientNetV2-S [42] is used as the keyframe encoder, and a UNet++ decoder [43] regresses the final depth map. For multi-scale fusion volumes, features from the first four residual blocks of ResNet18 [34] are extracted at $\frac{H}{4}$, $\frac{H}{8}$, and $\frac{H}{16}$ resolutions. The fusion MLP consists of four fully connected layers with 16 hidden units, expanding the 4-D prior cue to the cost-volume channel dimension *C*.

### Appendix A.4. Monocular Prior Extraction

The monocular model Metric3Dv2 (M3v) [24] is kept frozen during training. For each keyframe, M3v provides two pixel-wise priors: (1) a monocular depth estimate $\hat{d}$ and (2) an RGB-encoded surface-normal map $\hat{n}$. Since M3v does not provide confidence estimates, we compute a confidence prior $\hat{c}$ from the predicted depth by measuring its deviation from the valid inverse-depth range used during multi-frame matching. Pixels with $\hat{d}$ near or outside the predefined range [0,10] m receive lower confidence, while in-range predictions receive higher reliability scores. All monocular priors are computed once per keyframe and cached for subsequent iterations, introducing negligible runtime overhead.

### Appendix A.5. Normal-to-Scalar Projection

The surface-normal prior produced by the pretrained monocular model is provided in an RGB-encoded format $\hat{n}\left(u,v\right)\in R^{3}$. To integrate this prior efficiently into the hypothesis fusion pipeline, we convert the three-channel normal map into a scalar orientation cue using a luminance-based projection:

(A1) n(u,v)=0.299r+0.587g+0.114b.

This mapping preserves relative orientation variations while preventing over-parameterization in the fusion MLP.

### Appendix A.6. Sampling of Virtual Planes

For the loss based on virtual planes (Section 3.4), 15% of valid keyframe pixels are sampled per iteration. Triplets $\left(Q_{A},Q_{B},Q_{C}\right)$ are sampled from the keyframe only to avoid pose-induced noise. We set the maximum angular deviation to $\gamma =120^{\circ }$, the minimum deviation to $\delta =30^{\circ }$, and the minimum distance to ϵ=0.6 m.

### Appendix A.7. Dataset and Evaluation Protocol

To emulate realistic online mapping, reference frames are drawn only from preceding timestamps. All competing methods are evaluated under identical causal-view constraints.

## Appendix B. Additional Results

### Appendix B.1. Evaluation Metrics

We report five standard depth estimation metrics: AbsDiff, AbsRel, SqRel, $a_{10}$, and $a_{25}$. Let Ω denote the set of valid pixels (|Ω|=N), d(p) the predicted depth, and $d^{*}\left(p\right)$ the ground truth at pixel p∈Ω. The metrics are defined as follows:

(A2) $$\begin{array}{c} \mathrm{AbsDiff}=\frac{1}{N}\sum_{p\in \Omega }\left|d\left(p\right)-d^{*}\left(p\right)\right|\;, \end{array}$$

(A3) $$\begin{array}{c} \mathrm{AbsRel}=\frac{1}{N}\sum_{p\in \Omega }\frac{|d\left(p\right)-d^{*}\left(p\right)|}{d^{*}\left(p\right)}\;, \end{array}$$

(A4) $$\begin{array}{c} \mathrm{SqRel}=\frac{1}{N}\sum_{p\in \Omega }\frac{{\left(d\left(p\right)-d^{*}\left(p\right)\right)}^{2}}{d^{*}\left(p\right)}\;, \end{array}$$

(A5) $$\begin{array}{c} a_{x}=\frac{100}{N}|\left\{p\in \Omega :\max\;\left(\frac{d(p)}{d^{*}\left(p\right)},\frac{d^{*}\left(p\right)}{d(p)}\right)<1+\frac{x}{100}\right\}|\;,\;x\in \left\{10,25\right\}\;. \end{array}$$

Here, $a_{10}$ and $a_{25}$ denote the percentage of pixels within 10% and 25% relative error bounds. Lower is better for AbsDiff/AbsRel/SqRel; higher is better for $a_{x}$.

### Appendix B.2. MVSA on GMU

Table A1 reports the raw evaluation metrics of MVSA on the GMU dataset. Although data preprocessing was verified to be correct, the majority of predictions degenerated into nearly uniform outputs with no meaningful depth variation. As a result, the reported numbers are abnormally poor. Figure A1 further illustrates this issue: while some frames show valid depth structure, most collapse into a single-color map, highlighting the instability of MVSA under the GMU sensor configuration. For transparency, we include these results here but omit them from the main comparison tables.

**Table A1:** MVSA results on GMU. **Bold** indicates the best performance, and arrows (↑/↓) indicate that higher/lower values are better. The metrics collapse due to degenerate uniform predictions, demonstrating that MVSA is unsuitable for GMU. Reported here only for completeness.

Method	AbsDiff ↓	AbsRel ↓	SqRel ↓	$a_{10}$↑	$a_{25}$↑
MVSA	6.1457	5.6316	74.0462	1.1209	2.6884
